# Engaging One Health for Non-Communicable Diseases in Africa: Perspective for Mycotoxins

**DOI:** 10.3389/fpubh.2017.00266

**Published:** 2017-10-16

**Authors:** Carina Ladeira, Chiara Frazzoli, Orish Ebere Orisakwe

**Affiliations:** ^1^Environment and Health Research Group, Escola Superior de Tecnologia da Saúde de Lisboa – Instituto Politécnico de Lisboa (ESTeSL – IPL), Lisboa, Portugal; ^2^Grupo de Investigação em Genética e Metabolismo, Escola Superior de Tecnologia da Saúde de Lisboa – Instituto Politécnico de Lisboa (ESTeSL – IPL), Lisboa, Portugal; ^3^Centro de Investigação e Estudos em Saúde Pública, Escola Nacional de Saúde Pública, ENSP, Universidade Nova de Lisboa, Lisboa, Portugal; ^4^Department for Cardiovascular, Dysmetabolic and Aging-Associated Diseases, Istituto Superiore di Sanità, Rome, Italy; ^5^Toxicology Unit, Faculty of Pharmacy, University of Port Harcourt, Port Harcourt, Nigeria

**Keywords:** food safety, food security, immune system, risk assessment, risk management

## Abstract

The role of mycotoxins—e.g., aflatoxins, ochratoxins, trichothecenes, zearalenone, fumonisins, tremorgenic toxins, and ergot alkaloids—has been recognized in the etiology of a number of diseases. In many African countries, the public health impact of chronic (indoor) and/or repeated (dietary) mycotoxin exposure is largely ignored hitherto, with impact on human health, food security, and export of African agricultural food products. Notwithstanding, African scientific research reached milestones that, when linked to findings gained by the international scientific community, make the design and implementation of science-driven governance schemes feasible. Starting from Nigeria as leading African Country, this article (i) overviews available data on mycotoxins exposure in Africa; (ii) discusses new food safety issues, such as the environment–feed–food chain and toxic exposures of food producing animals in risk assessment and management; (iii) identifies milestones for mycotoxins risk management already reached in West Africa; and (iv) points out preliminary operationalization aspects for shielding communities from direct (on health) and indirect (on trade, economies, and livelihoods) effects of mycotoxins. An African science-driven engaging of scientific knowledge by development actors is expected therefore. In particular, One health/One prevention is suggested, as it proved to be a strategic and sustainable development framework.

## Mycotoxins Exposure and One Health (OH)

The burden of non-communicable diseases (NCDs) increasingly falls on the low- and middle-income countries and highlights the need for prevention of NCDs to be a part of development initiatives to reduce poverty and associated social and health inequalities. NCDs and novel (toxicant-related) zoonoses are linked with new issues in food safety, such as the environment–feed–food chain and toxic exposures of food producing animals (1). OH is the *joint effort of different discipline and sectors working at national, regional, and global level, to achieve the best possible health for communities, animals and the environment* (2). The OH concept acknowledges the web of links and interrelations that exist between human, animal, and environmental health. Broad institutional changes, implying transdisciplinary, multidimension, multisector, and multiactors approaches, and including transboundary harmonization and involvement of health and non-health sectors, are required for OH to become a widespread approach to health policy (3), both at local and global levels (4).

The role of mycotoxins has been recognized in the etiology of a number of NCDs. Mycotoxins are toxic secondary metabolites of fungal origin (e.g., *Aspergillus, Penicillium*, and *Fusarium* genera) and contaminate agricultural products and feeds before or under postharvest. Despite differences in contamination levels, exposure to mycotoxins is apparent globally: calculations show that approximately 24–50% of all the commodities produced globally, especially basic foodstuffs, can be contaminated by mycotoxins (5–7). In economically developed countries where food safety regulations are in place and climate is temperate [e.g., European Union (EU)], mycotoxins are a problem deserving continuous monitoring, control, and efforts to improve management. In many African countries, the public health impact of mycotoxins exposure is largely ignored even in face of rising incidence of liver cancer (8), esophageal cancer (9, 10), neural tube disorders (11), stunted growth (12–14), and other outcomes associated with mycotoxins (15). Moreover, mycotoxins in feeds and derivatives reduce livestock and crop production and influence or even impede export for safety reasons (6). In zootechny, economic losses due to animal consumption of mycotoxin [e.g., aflatoxins (AFs)] contaminated feeds are associated with reduced feed intake, feed refusal, poor feed conversion, diminished body weight gain, increased disease incidence (due to immune suppression), and reduced reproductive capacities (7, 16). Examples of mycotoxins of greatest public health and agro-economic significance include AFs, ochratoxins (OTs), trichothecenes (TCTs), zearalenone (ZEN), fumonisins (Fs), tremorgenic toxins, and ergot alkaloids (17–19). Differences in regulations exist between countries. In the case of AF, for instance, the EU sets limits for AFB1 and for total AFs (B1, B2, G1, and G2) in nuts, dried fruits, cereals, and spices. Limits vary according to the commodity, but range from 2 to 12 ng/g for B1 and from 4 to 15 ng/g for total AFs. There is also a limit of 0.050 ng/g for AFM1 in milk and milk products. Limits of 0.10 ng/g for B1 and 0.025 ng/g for AFM1 have been set for infant foods (20). US food safety regulations include a limit of 20 ng/g for total AFs (B1, B2, G1, and G2) in all foods except milk and a limit of 0.5 ng/g for AFM1 in milk. Australia and Canada set limits of 15 ng/g for total AFs (B1, B2, G1, and G2) in nuts, the same as the international limit recommended for raw peanuts by the Codex Alimentarius Commission (CAC).

Mycotoxins are substances with low persistence in the sense that they do not bioaccumulate. Some mycotoxins (e.g. aflatoxin, ochratoxin) are found as parent compound or their metabolites in milk and eggs. However, the main contribution comes from vegetable foods.

AFB1 contamination of feeds is a risk for the health of several farm animals, including fishes; milk is the only food of animal origin where a significant feed–food carryover may occur. A statutory limit (0.020 mg/kg feed) is established in Europe (21, 22).

Mycotoxins can enter the feed and food chains through direct or indirect contamination pathways. Direct contamination occurs when the food or feed becomes infected by a toxigenic fungus, with the subsequent formation of mycotoxins (23). Indirect contamination occurs when an ingredient has been previously contaminated by a toxigenic fungus and, even though the fungus has been eliminated during processing, mycotoxins remain in the final product (6).

The changing climate may increase the burden of mycotoxins contamination of feeds and foods globally and affect livestock production in terms of both food safety and security (24). The most common mycotoxins reported in Africa are AFs (43.75%) followed by Fs (21.87%), OTs (12.5%), ZEN (9.38%), deoxynivalenol (DON) (6.25%), and beauvericin (BEA) (6.25%) (25). Rampant and *in utero* AF exposure in some African countries, including Nigeria, has been found with hematological evidence (biomarkers) in at least 98% of the population (26). Following the approach of the environment–feed–food chain, OH strategies should be adopted in Africa for the prevention of mycotoxins exposure.

### Mycotoxins in African Staple Foods

Human ingestion of mycotoxins occurs mainly through contaminated plant food products or carryover in animal food products such as meat and eggs; noticeably, ingestion of mycotoxins’ metabolites occurs through dairy products (6). The most risky food commodities are wheat, maize, rice, beans, oleaginous seeds, cocoa, coffee, grapevine, wine, fruits, nuts, spices, and dried food (27–29).

In general, diet in Africa pivots on starchy staple foods/food products based in maize (especially corn fufu), cassava (*Manihot esculenta*) (e.g., water fufu and garri), plantain, rice, yams/cocoyams, and potatoes (30).

Specifically in Nigeria, carbohydrate intake, such as cassava, yam, and rice constitutes the main diet. Produced by cassava, garri is a roasted granular hygroscopic carbohydrate, popularly consumed by several millions of people regardless of ethnicity and socioeconomic class, making it the most common food product consumed in Nigeria. Garri can be consumed directly in the dry form with peanut, coconut, smoked fish, soaked in water or milk or boiled in water as porridge, popularly called “eba” and eaten with various types of African soups (31). Various groups of molds have been reported to be associated with garri during storage and distribution (32). When present, they can affect the nutritional quality of garri and lead to mycotoxin contamination in case of toxigenic species. OTA has been detected in cocoa and cocoa products in Nigeria (33), and very few reports of its incidence in other crops in Nigeria are available. A high level of 150 ng/g of the OTA was detected in maize (34) and moldy rice (35) from northern Nigeria. Ayejuyo et al. (36) found very low levels of OTA (0.0–2.1 ng/g) in 25 brands of imported rice marketed in Lagos metropolis. Data concerning mycotoxins levels in rice from Nigeria are sparse. Makun et al. (35) report the presence of AFB1, ochratoxin A (OTA), and ZEN in moldy rice, and other studies have been based on AFs (37). Ayejuyo et al. (36) assessed and found OTA in imported rice marketed in Lagos metropolis. Makun et al. (38) provided for the first time the mycotoxin profile of home-grown Nigerian rice with respect to seven of the most important mycotoxins worldwide, namely, AFs, OTA, ZEN, DON, T-2 toxin, fumonisin B (FB), and patulin (PAT). The study reports AFs detected in all samples, total AF concentrations ranging from 28 to 372 ng/g. ZEN (53.4%), DON (23.8), FB1 (14.3%), and FB2 (4.8%) were also found in rice, although at relatively low levels (38). The acceptable limits for ZEN, FBs, and DON are 30–200, <1,000, and 750–2,000 ng/g, respectively (39, 40). AF levels exceeding limits (10 ng/g) set by the 77 countries, including the EU, that regulate AFs were found in the homegrown Nigerian rice (38–40). OTA was found in 66.7% of the samples, with concentrations (134–341 ng/g) above the maximum levels (2–50 ng/g) in cereals for human consumption. Mycotoxins levels in some agricultural crops and foods in some African countries are shown in Table 1. The limit of quantification varies between 0.3 and 10 µg/kg depending on the mycotoxin.

**Table 1:** Mycotoxins levels (μg/kg) in the crops and foods in some African countries.

Country	Mycotoxin	Food stuffs	Concentration (μg/kg)	Reference
Mozambique	Fumonisin B_1_	Maize	159−7,615	Warth et al. (41)
	Fumonisin B_2_	Maize	27.7−3,061	
	Fumonisin B_3_	Maize	26.6−777	
	DON	Maize	116−124	
	DON-glucoside	Maize	12.6−32.5	
	NIV	Maize	20.2−45.9	
	ZEA	Maize	10.9−18.1	
	Citrinin	Maize	276−5,074	

Malawi	AF	Sorghum	1.7–3.0	Matumba et al. (42)
		Sorghum for thobwa drink	6.1–54.6	
		Sorghum for beer	4.3–1,138.8	

Botswana	AFs	Peanut	12–239	Mphande et al. (43)
Sudan	AFs	Sesame oil	0.2–0.8	Idris et al. (44)
		Groundnut oil	0.6	Elshafie et al. (45)
		Peanuts butter	21–170	
	AFB_1_	Sesame unpeeled	0.4–14.5	Kollia et al. (46)

Tanzania	FUMs	Maize	11,048	Kimanya et al. (47)
	AFs	Maize	158	

Tanzania and DR Congo	AFs	Maize	0.04–120	Manjula et al. (48)
Zambia	FUMs	Maize	20,000	Mukanga et al. (49)
	AF	Maize	0.7–108.74	Kankolongo et al. (50)

Uganda	AFs	Groundnuts, cassava, millet, sorghum flour	0–55	Kitya et al. (51)

Kenya	AFs	Animal feed and milk	>5	Kang’ethe and Lang’a (52)
		Maize	>20	Daniel et al. (53)
		Maize	1–46,400	Lewis et al. (54); Mwihia et al. (55)
		Peanut	0–7,525	Mutegi et al. (56)

Ethiopia	AFs	Shiro and ground red pepper	100–525	Fufa and Urga (57)
	AFs	Sorghum, barley, teff, and wheat	0–26	Ayalew et al. (58)
	OTA	Sorghum, barley, and wheat	54.1–2,106	
	DON	Sorghum	40–2,340	
	FUM	Sorghum	2,117	
	ZEA	Sorghum	32	

Nigeria	AFs	Rice	28–372	Makun et al. (38)
		Edible tubers “tiger nuts”	454	Adebajo (59)
		Edible tubers “tiger nuts”	10–120	Bankole and Eseigbe (60)
		Sorghum	10–80	Salifu (61)
		Dried yam	27.1	Bankole and Mabekoje (62)
		Dry roasted groundnut	52.4	Bankole et al. (63)
		Groundnut cake	20–455	Akano and Atanda (64)
		Peanut cake (*kulikuli*)	13–2,824	Ezekiel et al. (65)
		Corn-based snacks	12.0–30.0	Ezekiel et al. (66, 67)
		Nut-based snacks	0.0–6.0	
		Wheat-based snacks	0.0–50.0	
		Fin fish	1.05–10.00	Olajuyigbe et al. (68)
		Shell fish	4.23–5.90	
	OTA	Rice	134–341	Oluwafemi and Ibeh (69)
	AFs	Weaning food	4.6–530	
	OTA	Maize	0–139.2	Makun et al. (70)
		Millet	10.20–46.57	
		Sorghum	0–29.50	
		Sesame	1.90–15.66	
		Fonio (acha)	1.38–23.90	
		Cassava (garri)	3.28–22.73	

Ghana	AFs	Maize	0.7–355	Kpodo (71)
	Fs	Maize	70–4,222	Kpodo et al. (72)

Benin	AFs	Maize	5	Hell et al. (73)
		Chips	2.2–220	Bassa et al. (74)
		Dried yams	2.2–220	Mestres et al. (75)
		Cowpea	nd	Houssou et al. (76)

Benin, Mali, and Togo	AFs	Dried vegetables		
		Baobab leaves, hot chili, and okra	3.2–6.0	Hell et al. (77)

Burkina Faso	AFs	Groundnuts	170	Yameogo and Kassamba (78)
	DON	Maize	31.4	Warth et al. (41)
	ZEN	Maize	11.0−15.8	
	Citrinin	Maize	531−5,074	
	Alternariol	Maize	5.1−16.0	
	Altertoxin I	Maize	3.4−10.8	

South Africa	FUMs	Maize	222–1,142	Burger et al. (79)
	Fs	Compound feeds	104–2,999	Njobeh et al. (80)
	DON	Compound feeds	124–2,352	
	ZEN	Compound feeds	30–610	

Lesotho	ZEN	Sorghum beer	50	Gilbert (81)

*nd, not detectable; ZEN, zearalenone; DON, deoxynivalenol; AFs, aflatoxins; OTA, ochratoxin A; NIV, nivalenol*.

*Limit of quantification: DON = 10, NIV = 10, ZEN = 5, OTA = 0.3 µg/kg*.

### Mycotoxins in African Street Food

Common local street-vended snacks in Nigeria include beans cake (*akara*), roasted, dried and milled maize and groundnuts (*donkwa*), groundnut cake (*kulikuli*), fibrous powdery form of cassava (*lafun*), cheese curds (*wara*), and yam flour. Reports on mycotoxin contamination of these snacks have mainly focused on *Aspergillus* and *Penicillium* mycotoxins, such as AFs and OTA with scanty record on other fungal metabolites including *Fusarium* mycotoxins (66, 67). Snack samples made separately from corn, groundnut, and wheat were contaminated by total AFs concentrations at levels exceeding the limits for total AFs in foods (15 ng/g) as recommended by the National Agency for Food and Drug Administration and Control (NAFDAC), that is the regulatory body in Nigeria (66, 67). Noticeably, peanut cake, popularly called “*Kulikuli*,” is highly consumed due to its high protein and lipid content as well as its affordability by the many low- and middle-income people in sub-Sahara Africa (63). The AFB1 levels in *kulikuli* from different parts of Nigeria were about 200-folds more than the 10 µg/g NAFDAC limit and also higher than levels reported previously in peanut and peanut products (64, 82, 83). Rubert et al. (84) reported high levels of AFs (26 ng/g) in Nigerian baked coconut; α-zearalenol (α-ZOL) (54 ng/g) was found in coconut candy. Taken together street-vended snacks (cassava-, coconut- and groundnut-based types) in Nigeria seem contaminated by AFs. In Benin, Nigeria’s closest neighbor, AFB1 was detected in 93.3% of peanut cake samples at concentrations above the EU limit (85). The consumption of peanut cakes with high levels of AFB1 portends a public health concern since the consuming population is school-aged children and young adults in their active economical and reproductive age.

*Aspergillus flavus* and *Alternaria tenuissima* have been isolated from local Nigerian foods (86, 87). The 75–94.1% prevalence of nephrotoxic OTA at level (5 ng/g) regarded as unsafe by the EU in maize, that is a major component of weaning foods and animal feeds in Nigeria, makes its contamination by OTA a serious issue (70, 88, 89). Aflatoxigenic strains of *A. flavus* and *Aspergillus parasiticus* have been reported in peanut and peanut products in Africa (82, 90). *A. flavus* SBG is morphologically similar to *A. flavus* S-type strains and not only produces small sclerotia but also can synthesize large amounts of both AFs B and G. The SBG strain type has a more limited distribution and may be an important source of AF contamination in West Africa (91, 92). Perrone et al. (93) investigated the incidence of *Aspergillus* sect. *Flavi* and the level of AF contamination in 91 maize samples from farms and markets in Nigeria and Ghana. There was higher contamination of the farm samples than the market samples, suggesting that AF exposure of rural farmers is higher than previously estimated. High levels of AFs B and G and lower income of *A. flavus* SBG strains suggest that long-term chronic exposure to this mycotoxin are much higher health risk in west Africa than is the acute toxicity due to very highly contaminated maize in east Africa (93).

### Dietary Exposure to Mycotoxins’ Mixtures

Daily exposure to mycotoxins’ mixtures through consumption of single food sample is proven. Data on the co-occurrence of the principal mycotoxins in foods and beverages are increasing worldwide due to the availability and use of modern and sensitive LC–MS/MS methodologies suitable for simultaneous determination of mycotoxins and other fungal metabolites (94). The presence of mixtures of AFB1, OTA, and ZEN was reported in samples of breakfast cereals commercialized in Spain (94, 95). The study conducted by Solfrizzo et al. (94) on mycotoxins exposure in southern Italy confirmed the presence of DON and OTA in almost all urinary samples. In this study, 6% of urine samples contained AFM1, i.e., a metabolite of mycotoxin mainly found in maize (AF M1 is not present in Maize) and derivatives although these products are not staple foods in Italy where they are consumed as chips, polenta, popcorn, beer, cornflakes, snacks, muesli, and mixed cereals. From a risk assessment stand point, the co-occurrence of mycotoxins is very important though vaguely understood: indeed, recent *in vitro* data highlight potential additive or synergistic interactions (96–99). Notwithstanding this, also in Europe there are few published studies on the co-occurrence of mycotoxins [e.g., Ref. (100, 101)]. Co-contamination with AFs, OTA, and ZEN is very common in Nigeria, and up to five mycotoxins were detected in a single rice sample; AFs (B1, B2, G1, and G2) were found in all samples (38). The presence of AFs and OTA in this Nigerian staple food at levels exceeding the limits set by international regulatory bodies along with the co-occurrence of other toxicants with possible toxic synergistic effect made the studied rice sample unsuitable for human and animal consumption and raise national public health concerns (38). Kimanya et al. (102) confirmed co-occurrence of AFs with DON and Fs from maize based meals in northern Tanzania. In a survey of mycotoxins in traditional maize based opaque beers in Malawi, it was estimated that consumption of 1.0–6.0 L of this local beverage results in a daily FB1 and FB2 exposure of 29–174 µg/kg body weight (bw)/day [i.e., >provisional maximum daily intake of 2 µg/g bw/day set by the Joint FAO/WHO Expert Committee on Food Additives (JECFA)] and AF exposure of 1.5–9.0 µg/kg bw/day for a 60 kg adult (103). This is of significant public health importance since this singular source alone can add to the body burden due to AFs and Fs dietary exposure among beer consumers (103). OTA, ZEN, DON, NIV, and other less reported mycotoxins such as citrinin, alternariol, cyclopiazonic acid, sterigmatocystin, moniliformin, BEA, and enniatins were detected in various food samples from Burkina Faso and Mozambique (41). The quantification of at least 28 toxic fungal metabolites in a single sample strongly suggests the huge variety of mycotoxin co-exposure in Africa (41).

Ngoko et al. (104) report 50–26,000 ng/g Fs, 100–1,300 ng/g DON, and 50–180 ng/g ZEN in maize samples from Cameroon. Detectable levels of AF ranged between 5.2 and 14.5 ng/g in other widely consumed foods in Cameroon, namely, cassava balls and cassava pellets (105). A simultaneous occurrence of mycotoxins (FB_1_ 41%, AF 51%, ZEN 57%, DON 65%, and OTA 3%) in human food commodities from Cameroon has also been reported (80). In another study from Cameroon, total AF levels exceeded the maximum limits of the European Commission (EC) regulations (30). Taken together, the widespread nature and high levels of multiple mycotoxins occurring in staple foods suggest high exposure levels that could have severe health implications in sub-Sahara Africa.

AFM1 in human breast milk is an important health risk for infants (16). The chronic intake of AF contaminated food could increase stillbirths and neonatal mortality, immune suppression with increased susceptibly to infectious diseases such as pneumonia, stunting of growth (33), and HIV/AIDS (106). In many countries, because animals are usually milked individually at the household doorstep mycotoxins consumption can be very high (107–109). Although the minute of mycotoxins through food of animal origin may be seemingly innocuous in the general population, vulnerable groups may not be spared, especially the genotoxic carcinogens such as AFs.

### Additional Exposure Route: Mycotoxins in African Indoor

Inhalation of contaminated airborne aerosols can represent an additional route of mycotoxin exposure. Nowadays, people spend about 90% of their time in indoors environment due to working or resting (31). However, in many parts of the world, homes, schools, and workplaces are contaminated with airborne molds and other biological contaminants (110, 111).

Mycotoxins can be found in airborne particulates of environments where susceptible commodities are treated, such as warehouses, harbors, laboratories, and specific occupational settings where products/materials that are commonly contaminated (e.g., waste, feed, and animal production) are handled (112–115).

Poultries fungal burden is mainly affected by the kind of litter applied in pavilions (112), whereas in swine it is mainly affected by the feeding operations due to feed fungal contamination (113, 114). Waste management industries pose another challenge regarding workplaces fungal contamination, in waste water treatment plants and in solid waste management industries the main source are the waste water and the waste that need to be treated (115).

Moreover, the presence of mycotoxins in domestic households as a consequence of inappropriate hygiene conditions has been demonstrated, with immunosuppressive effects due to the inhibition of phagocytosis and of alveolar macrophage functions (27). Children, elderly, patients on immune suppressants, and with respiratory diseases are more susceptible to contamination by indoor fungi (110). *A. flavus* has been isolated from indoor environment like hospitals in Nigeria (116, 117). Although the presence of indoor fungi by mold contamination is related with dampness of the indoor environment and swampy locations, researches have indicated fungal presence as well in houses without these characteristics (111). The highest isolation rates (*Rhizopus* sp., for instance) were achieved from high residential density areas, probably an effect of overcrowding, poor sanitation and high arthropod infestation. Factors such as absence of basic facilities for drainage and waste disposal and dumps in proximity of residential homes do favor indoor mold contamination (118, 119).

## Risk Assessment

Mycotoxins are metabolized in liver and kidneys and also by microorganisms in the digestive tract (7). Chemical structure and toxicity of mycotoxin metabolites excreted by animals or found in their tissues are different from the parent molecule. Toxicity depends of factors such as type of toxin, dose ingested, duration of exposure, age, and sex (29). The WHO (120) estimated that AFs were responsible for nearly 20,000 deaths each year, 3,000 of them on the African continent. The International Agency for Research on Cancer (IARC) classified AFB1 in group 1 “*carcinogenic to humans*.” AFB1 is the most potent natural carcinogen and is usually the major AF produced by aflatoxigenic strains. The no observed-adverse effect level is not applied for genotoxic carcinogens, therefore no threshold is assigned to AFB1. In particular, AFs are potent hepatotoxins. Chronic exposure to small doses of AF for prolonged periods (e.g., through the diet) has been associated with human hepatocellular carcinomas, which may be compounded by other carcinogens, such as hepatitis B virus. Hepatocellular carcinoma (HC) is the third most common cause of death from cancer in Africa (121). Approximately 250,000 deaths are caused by HC in sub-Saharan Africa annually and can be attributed to risk factors such as high daily intake (1.4 µg) of AF and high incidence of hepatitis B (17, 19). As well as causing liver cancer, AFs have been associated with other health problems in people such as stunting in children and immune suppression (16). Chronic exposure to AFs is associated with impaired immunity and malnutrition, therefore also with malaria and HIV/AIDS (21, 22, 122, 123). A study in Ghanaian adults reported that AFs could cause impairment of human cellular immunity that could decrease resistance to infections (19). Kwashiorkor, a disease usually considered a form of protein energy malnutrition, has long been linked to AF exposure, along with chronic gastritis and childhood cirrhosis (14, 124). Acute exposure to large doses (>6,000 μg) may precipitate severe acute liver injury with high morbidity and mortality (125). Symptoms of acute toxicity include reduced liver function, derangement of blood clotting mechanism, icterus (jaundice), and a decrease in essential serum proteins synthesized by the liver. Acute AF exposures have been associated with epidemics of acute toxic hepatitis in Africa with death rates ranging from 10 to 60% (6, 17). Other general signs of aflatoxicosis are edema of the lower extremities, abdominal pain, and vomiting. An outbreak of acute aflatoxicosis in Kenya in 2004 caused 125 deaths among 317 people that consumed AF contaminated maize (92).

Aflatoxin M1, OTA, and FB1, FB2 are classified in group 2B “*possibly carcinogenic to humans*.” Chronic ingestion of Fs has been linked as possible risk factor for the occurrence of esophageal cancer in areas, such as the former Transkei region of South Africa, where Fs exposure from contaminated maize is high (126). There is a specific *p53* codon 249 mutation in the plasma of liver tumor patients from West Africa (Gambia) after exposure to AFs (127, 128). In a study of HIV and hepatocellular and esophageal carcinomas, related to consumption of mycotoxin-prone foods in sub-Sahara Africa, the relation between cancer and food suggested that Fs contamination rather than AF is the most likely factor in maize promoting HIV (129). OTA could also be associated with immunotoxic and neurotoxic effects (29).

Other mycotoxins, i.e., PAT, ZEN metabolites, some TCTs, in particular T-2 toxin, nivalenol (NIV), and DON, are considered by IARC as “*not classifiable as to its carcinogenicity to humans*” (group 3).

With special emphasis on infertility, that is an ongoing global reproductive health problem, also in Africa, *in vivo* and *in vitro* studies have shown that ZEN and metabolites [α-ZOL and β-zearalenol (β-ZOL)], DON, OTA, and AFB1 adversely affect fertility by arresting steroidogenesis. Exposure to these mycotoxins precipitate deleterious effects on the spermatozoa, Sertoli and Leydig cell function, oocyte maturation, and uterine and ovarian development and function in *in vivo, ex vivo*, and *in vitro* experimental models (130–134). Mycotoxins can induce oxidative stress and result in damage of sperm DNA (135), reduced fertilization rates and embryo quality (136). Mycotoxins have also been implicated as endocrine disruptors altering the steroid hormone homeostasis and interfering with receptor signaling (137–140). Concentrations of AFB1 significantly higher in the semen of infertile men than in controls (semen of fertile) have been reported by Ibeh et al. (141), thus suggesting that exposure to AFB1 could be a causative factor in male infertility in Nigeria. At least 50% of infertile men with high seminal concentrations of AFB1 had a greater percentage of abnormalities in sperm count, motility and morphology compared with the fertile men (10–15%) (141). These observations were comparable to male rats fed with AFB1 contaminated feeds (8.5 µg/g of feed) for 14 days (141). Similarly, semen and blood levels of AFB1 which ranged from 700 to 1,392 ng/mL and exceeded the WHO recommended level have reported in infertile men attending the infertility clinic in Nigeria (142). The high prevalence of male infertility in Africa (20–35%) (143–146) as well as the declining sperm count (147) motivate reproductive health experts in investigating the role of mycotoxins (148). Since endocrine disrupting chemicals are known to cause endometriosis, premature ovarian failure, and polycystic ovary syndrome, mycotoxins may also be involved in female reproductive disorders (149).

### Markers and Biomarkers

Mycotoxins are measured in feeds, food, air, or other environmental samples for environmental monitoring purposes, whereas the presence of adducts and metabolites are assayed in human or animal tissues, fluids, and excreta for biological monitoring (150). A challenge in the field of internal exposure assessment is to develop accurate and reliable biomarkers. The biomarker approach is a promising tool for measuring toxin-mediated biological perturbations or the amounts of mycotoxins present in the matrix (28). In molecular epidemiology, it is possible to demonstrate the association between putative carcinogens and specific cancers (150). Biological markers of AFs, OTA, and Fs exposure have attracted the attention for mycotoxin biomonitoring studies. However, while AFs and OTA biomarkers have been successfully applied and validated over the last decade, large drawbacks remain to find a suitable Fs biomarker (28).

Biomonitoring of AFs can be done by quantifying AF metabolites in blood, milk, and urine. Indeed, the first studies in which biomarkers where used to determine human exposure to food pollutants involved AFB1. In these studies, correlation between AFB1 intake and urinary AFM1 excretion was statistically achieved and the exposure biomarker validated. The mean urinary AFM1 level in Cameroon (30) was similar to that observed in adults in Ghana (range: nd–0.115 µg/L) (151) and fully weaned that of Guinean children (152). A similar range was observed among pregnant women in Egypt (0.004–0.409 µg/g creatinine) (153). Ghana and Guinea are recognized as high-risk regions for AF exposure, whereas Egypt is regarded as moderate when compared with sub-Saharan Africa (152, 154–156). The estimates of tolerable daily intake of several mycotoxins are exceeded in Africa (30). In a pilot, cross-sectional and correlational study conducted in eight rural communities in northern Nigeria to investigate mycotoxin exposures in volunteers, urinary biomarker levels were correlated with mycotoxin levels in foods consumed the day before urine collection in all age categories, suggestive of chronic (lifetime) exposures (10). In the urines with detectable AFM1, it was estimated that the mean intake of AFB1 was 0.67 ng/kg bw/day (max = 2.5 ng/kg bw/day). Higher AFM1 urinary levels have been detected in children from Sierra Leone children (157).

Albumin-bounded AFB1 and AFB1-DNA adducts in urine have also been explored for exposure assessment studies (28). Numerous studies have shown that carcinogenic potency is highly correlated with the extent of total DNA adducts formed *in vivo* (150). Excreted DNA adducts and blood protein adducts can also be monitored: the AFB1-N^7^-guanine adduct represents the most reliable urinary biomarker for AF exposure but reflects only recent exposure (158). High AF-albumin adduct levels in maternal blood, cord blood, infant blood, and children’s blood have been associated with poorer growth indicators and impaired markers of human immunity as shown by lower levels of secretory immunoglobulin A in saliva of Gambian children (159, 160). High levels of AFB1-albumin adducts were associated with low percentages of certain leukocyte immune phenotypes in Ghana (161). The chronic/dietary exposure to AF is evident from the presence of AFM1 in human breast milk (162) and umbilical cord blood samples (163), with serious implications for the next generation (109). Home-grown maize contamination led to arguably the largest fatal aflatoxicosis outbreaks in rural communities of Kenya, in which AF-albumin adducts were independently confirmed in the exposed (164). In another study from Kenya, wasting in children was related to consumption AF contaminated flour (165). In Ghana, low birth weight was shown to have an association with mothers’ AF-albumin adduct levels (166). There is a dose-dependent decrease in height and weight for age in AF exposed children in a study carried out in Togo and Benin in West Africa (123, 167).

Mycotoxin-producing molds have lately been found to infect the intestinal tract to cause leaky gut, thus exerting important immunosuppressive activity, and produce neurotoxins (168). OTA, that has nephrotoxic, hepatotoxic, immunotoxic, and genotoxic effect and induces carcinogenicity, teratogenicity, and mutagenicity, has also been seen to cause dysregulation of several gene expression including the upregulation of SOX9 (169), i.e., a gene involved in the development of the male phenotype and has been detected in autistic cases (170). It has recently been posited that single nucleotide polymorphisms in NLGN4X 3′UTR and illegitimate microRNA-inducing OTA could be a possible biological mechanism reflecting the gene–environment interaction in patients without causative mutations (171, 172) and suffering from dysbiosis and leaky gut (173). Although there seem to be no published data on population-based estimates of prevalence of pervasive developmental disorders from African region, the prevalence of autism spectrum disorder (ASD) among children with developmental disorders in Egypt and Tunisia has been documented as 33.6 and 11.5%, respectively (174, 175). The ASD is an increasing neurodevelopmental disorder with a broad phenotype, appearing by 3 years of age: it often shows comorbid situations, such as mental retardation, epilepsy, and recurrent gastrointestinal abnormalities. In Nigeria about 0.9% of the children under the age of 3 years manifested neurodevelopmental delays in a recent survey (176). It is even feared that this value may be higher considering late diagnosis (176). Like most aspects of ASD, the mycotoxin impact on this prevalence remains unknown.

Human health risk assessments of Fs hinge on maize consumption. Maize consumption can be <10 g/person/day in various European countries, but up to 400–500 g/day in rural Africa (177), with a 90 percentile value of over 700 g/person/day (178). The implication of this socio-geographical dietary variation with respect to attaining the provisional maximum tolerable daily intake (PMTDI) of 2 ng/g bw/day of Fs is enormous. Whereas a European consumer at an assumed bw of 60 kg would need to consume 10 g maize at an Fs contamination level of 12,000 ng/g, an African who consumes 500 g/day would exceed the PMTDI if the contamination level was above 240 ng/g (179). The detrimental effects of Fs on the developing fetus and young infants are now known from both experimental and epidemiological researches. Transkei region in South Africa and Tanzania where Fs exposure is high is known to have elevated incidences of neural tube defects and growth retardation (180, 181). Fs interfere, *via* depletion of sphingolipids, with the folate receptor, thus inhibiting the uptake of folate and eventually leading to cellular folate deficiency and neural tube defects (182), that can be prevented in experimental animals by folate supplementation (183).

## Perspectives for Risk Management in West Africa

Many African countries have some mycotoxin regulations but only for AFs (or a few other mycotoxins) in specific foods, or no regulations at all. Even when standards are in place, severe mycotoxin-poisoning outbreak occurs in Africa (92). Indeed, good practices and recommendations for the field management of risk of mycotoxins occurrence would be strategic for investment of public, non-governmental organization, and private funds at the scale of the subsistence farmer, the smallholder, and through to a more advanced value chain (184).

The multiplicity of origins of fungal infections implies that strategies for prevention of mycotoxins contamination must be applied at an integrative level along all the food production chain. There are three steps of intervention that must be of concern: prevention (i) before any fungal infestation, (ii) during the period of fungal invasion of plant material and mycotoxins production, and (iii) when agricultural products have been identified as heavily contaminated (7, 185).

Risk mitigation practices cover pre- and postharvest:

(i) *Predictive models*. Weather conditions (e.g., hot and humid tropical climate that favors fungal proliferation) are the most influential parameter on mycotoxins contamination and fungal infection and growth (186, 187). Predictive models for mycotoxins occurrence based on regional weather data would be a valuable tool to estimate the risk of contamination (188). In a study that examined AF exposure in pregnant Gambian women having staple food in refined white rice, millet, or maize with groundnut sauce, AF exposure throughout pregnancy was found, with higher levels in the dry season. Women in later stages of pregnancy showed higher levels of AF-albumin adducts than those in earlier stages of pregnancy in the dry season (189).
(ii) *Preharvest interventions*. Good agricultural practices such as sanitation, early sowing date, balanced nitrogen fertilization, moderate plant density, breeding for resistance to drought, insect pest damage or fungal infections, biological control, early harvesting, and moisture levels and proper handling during harvesting. An integrated program involving plant maturation, nutrition, and insect control is crucial (9, 185), along with proper and timely crop rotation, tillage, and fungicide administration (7, 150). Biogeographical agricultural models of cultivated plants could also be useful.
(iii) *Postharvest strategies* (transportation, marketing, and processing). Control of factors such as temperature, humidity, pH, packaging, cross contamination by practices like sorting and complete drying decrease contamination during storage (46). In case of toxin manifestation, measures are required that act specifically against certain types and groups of toxins (7, 150).
(iv) *Detoxification strategies* for contaminated feeds are studied to reduce or eliminate the adverse effect of mycotoxin. The addition to the animal’s diet or the treatment of contaminated feeds with mycotoxin-binding agents may be useful to protect animal health and avoid milk contamination by the carcinogenic AFM1 metabolite. However, mycotoxin binders may impact animal health, e.g., by interfering with the absorption of nutrients or medications (7, 190). Traditional techniques that could reduce/detoxify mycotoxins during food processing are studied (191).
(v) *In house protective practices*, such as proper food storage, dietary diversity—where possible—, and vaccination against HBV to prevent the synergism of AF exposure and chronic HBV infection in liver cancer risk (7, 95, 150, 192–194). Significant building blocks for mycotoxins risk management do exist in West Africa, such as the following:
  - *Surveillance and monitoring of environmental/food matrices experiences*. Biomonitoring of mycotoxins in biological fluids such as blood or urine is useful to generate reliable information on internal exposure at individual level compared with dietary assessments (10). Validated *biomarkers of exposure* are available, such as urinary metabolites, DNA, and protein (albumin) adducts (15, 192). The OTA levels found in Nigerian-grown rice and maize are within the lower limits of concentrations (200–1,000 ng/g) that have been linked to porcine nephropathy in Bulgaria (195). There has been a speculation about the contribution of OTA to raise the incidence of chronic renal diseases in Nigeria in conjunction with malaria, hypertension, and diabetes conditions. Poor record of renal *registry* in Nigeria has hampered the tracking of chronic renal disease; however, available hospital data revealed that chronic renal failure accounts for about 10% of medical admissions in Nigeria, and extrapolating this, puts the frequency figure between 200 and 300 patients per million of population (196).
  - *Application of biomarkers*. In a pilot study using multi-urinary biomarkers among rural residents in northern Nigeria, Ezekiel et al. (10) detected mycotoxin in all age categories. Their observations suggest chronic/lifetime exposures, and some exposures were higher than the tolerable daily intake. The study developed in Cameroon by Abia et al. (30) used for the first time in Africa a novel multi-mycotoxin assay utilizing LC–MS/MS to determine the frequency of occurrences and levels of several mycotoxins, or their metabolites in urine.
  - *Experiences of total diet studies (TDSs)*. Dietary intake estimate should include data on consumption of raw and processed foods (100) to assess average dietary exposure and identify excessive consumer subgroups. TDSs are often used as a risk assessment tool to evaluate exposure and—when performed periodically- exposure trends in the general population and (more vulnerable consumers such as children or diseased subjects, or higher consumers) high-risk subgroups. TDSs differ from traditional food monitoring in two major aspects: (i) chemicals are analyzed in food in the form in which it is consumed and (ii) cost-effectiveness, because composite samples (more ingredients grouped) after kitchen processing are analyzed. As made by European participants in the SCOOP [Scientific Cooperation on Questions relating to Foods (197)] exercises, African countries could group by region and collect, and harmonize knowledge on the status of mycotoxins contamination of raw material and food products (197). Preliminary experiences of TDSs do exist in West Africa, along with its methodology and methods (198).
  - *Seminal governance framework based on OH*. OH integrates efforts for building a governance national strategy based on the linked and mutually supported protection of environment, farm animals and human well-being (199).
  - *Seminal toxico-vigilance (TV) system*. The TV system aims at updating (and harmonizing) registers on information on incidence of poisoning in communities (200).
  - *Risk assessment and advices for food regulations*. Mycotoxins regulations have been established in about 100 countries, out of which 15 are African, to protect the consumer. As in the case of Europe (the European Food Safety Authority), an African independent body could be established with the task of independent science-based risk assessment on food and feed. So far, the JECFA, that is an international committee administered jointly by FAO and WHO, serves as an independent scientific committee which performs risk assessment and provides advice to FAO, WHO, and the member countries of both organizations. The requests for scientific advice are for the main part channeled through the CAC in its work to develop international food standards and guidelines under the Joint FAO/WHO Food Standards Programme.

### African Turning Point on Mycotoxins

Mycotoxins are now recognized as major cause of food intoxications in SSA. Many economically developing countries have realized that reducing mycotoxins level in foods will not only reduce financial burden on health care but also confer international trade advantages such as exports to more attractive and remunerative markets. Moreover, reducing mycotoxins level means facing lowered animal production, lowered yields in agriculture, and lower market value (5, 7, 17).

The study from Somorin et al. (101) concerning the co-occurrence of AFs, OTA, and citrinin in egusi melon seed from Nigeria is one of the examples to explain the basis for increasing border rejection of melon seed consignments from Nigeria to EU as highlighted in the European Rapid Alert System for Food and Feed (RASFF) (201). This led to the enactment of legislation which mandates that 50% of consignments of egusi and their derived products from Nigeria be checked before being allowed entry into the EU (202, 203).

**Figure 1:**
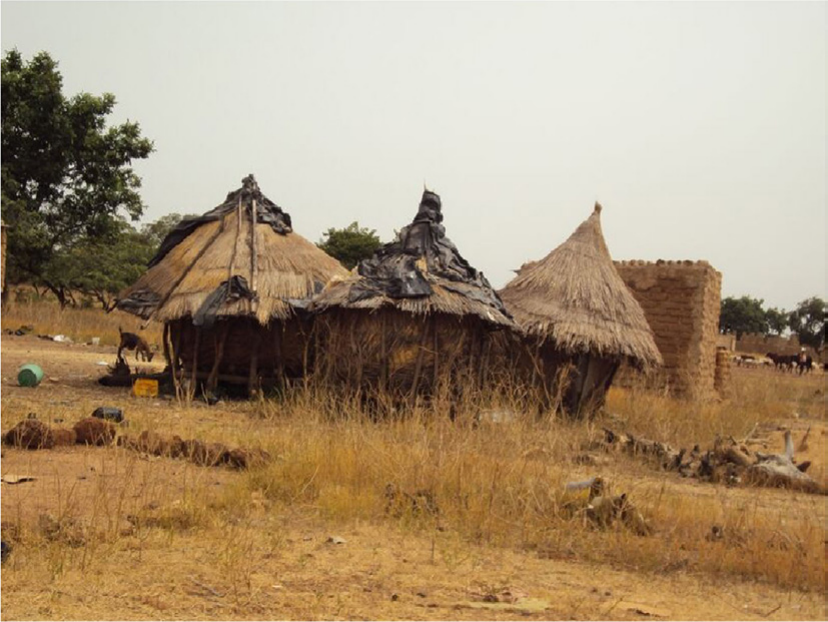
Granaries in Burkina Faso, 2012 (courtesy of Ilaria Proietti, NOODLES Alliance).

**Figure 2:**
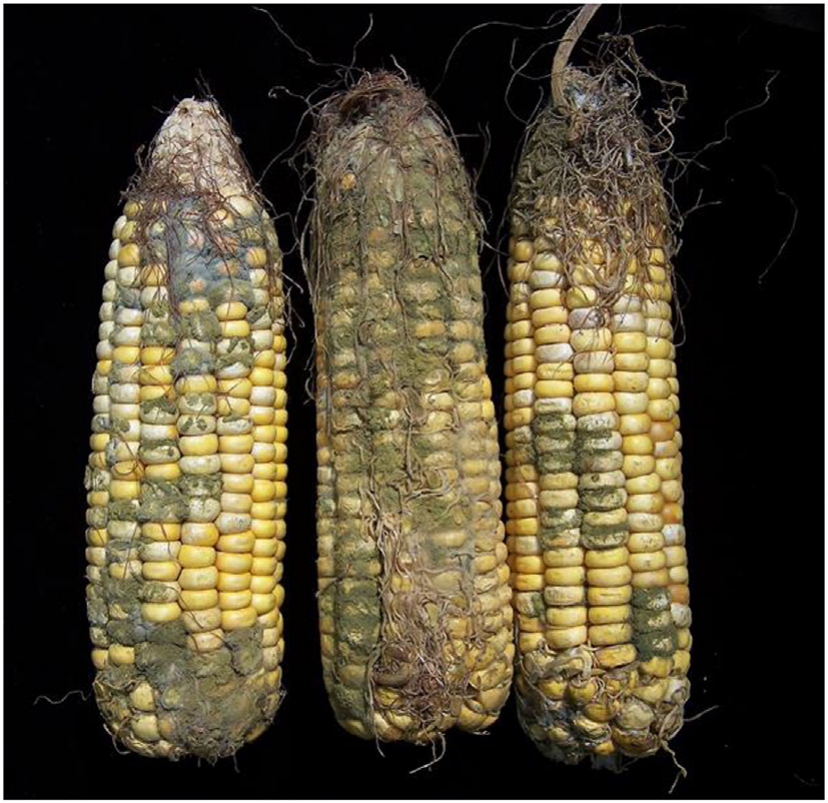
Photograph of mycotoxin contaminated food: reproduced from Environmental Health Perspectives; September 2013, Volume 121, Issue 9, doi:10.1289/ehp.121-A270.

Pivoting on what has already started in Africa, mentioned “building blocks” deserve strengthening and improvement. Based on the OH approach, mycotoxin reduction and control are dependent on the concerted efforts of all actors and stakeholders along the food production chain. We highlight here:

- *Political will* to address mycotoxins exposure and support capacity for testing commodities, which determines whether requirements can be enforced (162). As in the General Food Law issued by the EC, that clearly describes the food safety framework in the EU, including the role and responsibilities of the different parties involved from farm to fork, a envisaged African general food law could have a hierarchic and network character (21).
- *Strengthened laboratory capacities*, including efficient, cost-effective sampling, and analytical methods. Indeed, scientific research is moving toward reliable but cost-effective and sustainable user-friendly techniques for the acquisition of analytical data under field conditions and environmental stress (204).
- *Nationwide surveillance and regular monitoring* capacities by increased food and feed inspections (200).
- Established *early warning systems* as well as *risk management systems* allowing timely corrective actions and avoiding both food losses and waste (205).
- *Training and empowerment of farmers* and food producers on the good agricultural and good management practices. Indeed, communities are the foundation of Public health (205).
- *Improvement of facilities*. Many African countries do not have the infrastructures to prevent and control food contamination (e.g., Figure 1): science could give low cost solution to long lasting problems of infrastructures.
- *Consumer awareness and education*. According to Ezekiel et al. (65), at least 85% of the consumers of *kulikuli* in Nigeria are not aware of the risk of AF contamination of vended peanut cake. Consumers should prefer food producers adopting good practices.
- *Dissemination* of information *via* national media (radios, television, newspapers and magazines, and town hall meetings) and the web (206).
- *Food processors or industry* should contribute to an improved economic sustainability and enhanced international trade [see, e.g., reflections in Ref. (207)].
- *The “luxury” of choice* Figure 2. In countries where populations are facing starvation or where regulations are either not enforced or non-existent, chronic intake of AF may occur liver cancer incidence rates are 2–10 times higher in economically developing countries than in economically developed ones. Unfortunately, strict limitation of AF contaminated food is not always an option. A joint FAO/WHO/United Nations Environment Programme Conference report stated that in some economically developing countries, where food supplies are already limited, drastic legal measure may lead to food security problems, e.g., lack of food and excessive prices. It must be remembered that people living in these countries cannot exercise the option of starving to death today to live a better life tomorrow (150).

The following are some more aspects that deserve deep attention:

- *Risk analysis* is increasingly recognized as an essential component in modern science-based food safety systems and plays a growing and important role in guiding food safety authorities. Informed by the risk assessment process, risk management in its broadest sense involves the consideration and implementation of food policy options, while taking due cognizance of tolerable levels of risk. Risk communication involves the interchange of information concerning risk and its perception among all stakeholders in food safety, including policy makers, industry, and consumers (208, 209).
- *Risk to benefit assessment*. Interestingly, some of the food items that are prone to mycotoxins contamination are component of healthy diet. Based on RASFF reports, the most predominant category of mycotoxins is AF in pistachios, peanuts, almonds, hazelnuts, and Brazil nuts. OTA occurs mainly in beverages (raw coffee and derivatives, cocoa powder), fruit and processed fruits (mainly raisins/sultanas and figs), spices and condiments (mainly pepper), vegetables, cereals, and other crops (202). Models for risk to benefit assessment are increasingly available (210).

## Conclusion

Operationalization of OH for mycotoxins can shield population from direct (on health) and indirect (on trade, economies, and livelihoods) effects of mycotoxins. Mycotoxins effects on public health and economy in Africa are not directly measurable, though its existence is indicated by environmental, toxicological, and clinical data. The contamination of food and feed by mycotoxins represents a serious health problem as well a considerable economic obstacle in African countries, where the trade balance is based on the exportation of commodities. Nowadays, the poorest regions of the world have neither the infrastructures to prevent and control food contamination, nor the luxury to allow the rejection of contaminated food. Operationalizing mycotoxins in the OH frame is useful to build a risk management frame that is sound and understandable in terms of empirical observations by local institutional stakeholders expected to issue risk management programs in Africa. Indeed, governance schemes for early prevention of toxic exposures deserve inclusion in development initiatives.

## Author Contributions

CL, CF, and OO contributed equally from the literature search, write-up, and revision of this article.

## Conflict of Interest Statement

The authors declare that the research was conducted in the absence of any commercial or financial relationships that could be construed as a potential conflict of interest.
